# Modulation of the PD-1/PD-L1 axis and tumor immunity by metals and metalloids: mechanistic insights and human health implications

**DOI:** 10.3389/fimmu.2026.1862118

**Published:** 2026-06-05

**Authors:** Zhenyu Lu, Guangcan Chen, Jingle Qiu, Xia Huo, Xijin Xu

**Affiliations:** 1 Laboratory of Environmental Medicine and Developmental Toxicology, Shantou University Medical College, Shantou, Guangdong, China; 2 Department of Digestive Surgery, The First Affiliated Hospital of Shantou University Medical College, Shantou, Guangdong, China; 3 Laboratory of Environmental Medicine and Developmental Toxicology, Guangdong Key Laboratory of Environmental Pollution and Health, School of Environment, Jinan University, Guangzhou, Guangdong, China; 4 Department of Cell Biology and Genetics, Shantou University Medical College, Shantou, Guangdong, China

**Keywords:** cancer immunotherapy, immune checkpoints, immunotoxicity, metal exposure, metals and metalloids, tumor immune microenvironment, tumor immunity

## Abstract

Immune checkpoints maintain immune homeostasis, but their dysregulation can impair antitumor immunity and promote tumor immune evasion. Accumulating evidence indicates that metals and metalloids are important modulators of immune checkpoint signaling; however, their underlying mechanisms and broader implications for human health have not been systematically synthesized. In this review, we conducted a comprehensive search of PubMed, Web of Science, and Scopus through March 2026 to identify peer-reviewed studies examining metal- and metalloid-associated changes in immune checkpoint expression or function in human, animal, and experimental models. Current evidence suggests that metals and metalloids modulate immune checkpoints in a highly context-dependent manner, with the strongest support for effects on the PD-1/PD-L1 axis, whereas evidence for other checkpoints remains limited. Mechanistically, these effects converge on remodeling of the tumor immune microenvironment, oxidative stress, hypoxia signaling, inflammatory signal transduction, and tumor suppressor and protein homeostasis pathways, thereby influencing T-cell exhaustion, tumor immune evasion, and responses to immune checkpoint inhibitors (ICIs). Emerging clinical evidence further suggests that trace-element status, particularly zinc- and copper-related measures, may have value as candidate biomarkers in immuno-oncology. However, most evidence remains preclinical, and direct human evidence linking metal- and metalloid-associated checkpoint dysregulation to systemic adverse health outcomes or clinically actionable interventions is still limited. Overall, this review connects exposure biology with cancer immunology and identifies metal- and metalloid-associated immune checkpoint modulation as a plausible contributor to altered tumor immunity and a priority for future translational and population-based research.

## Graphical Abstract

**Figure:**
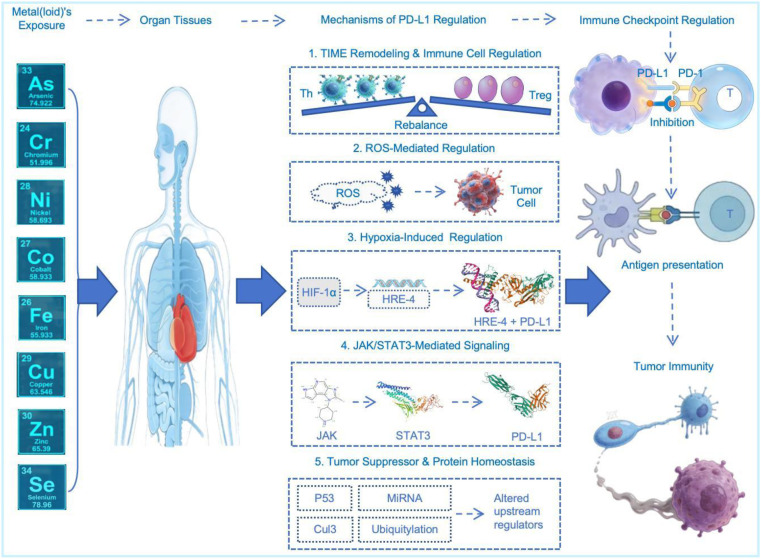

## Highlights

- Immune checkpoints are context-dependently modulated by metals and metalloids.
- Immune checkpoints are regulated via several pathways.
- Metal/metalloid imbalance disrupts immune checkpoints and ICB efficacy.
- Zn/Cu-related measures may serve as exploratory biomarkers for ICB therapy.

## 1 Introduction

Immune checkpoints are central regulators of immune homeostasis and antitumor immunity (1). Among these pathways, the programmed cell death protein 1 (PD-1)/programmed death-ligand 1 (PD-L1) axis is the most extensively studied and clinically validated immune checkpoint pathway. In parallel, other co-inhibitory immune checkpoints, including cytotoxic T-lymphocyte-associated protein 4 (CTLA-4), lymphocyte activation gene 3 (LAG-3), T-cell immunoreceptor with immunoglobulin and immunoreceptor tyrosine-based inhibitory motif domains (TIGIT), and T-cell immunoglobulin and mucin-domain-containing 3 (TIM-3), have also emerged as pivotal next-generation immunotherapeutic targets. In cancer, dysregulated checkpoint signaling promotes T-cell exhaustion, immune suppression, and tumor immune evasion (1), whereas antibodies targeting PD-1 or PD-L1 have produced substantial clinical benefit in multiple malignancies (2). However, clinical responses to ICIs remain highly heterogeneous, indicating that additional biological modifiers of checkpoint signaling need to be clarified.

While the strongest evidence currently centers on the PD-1/PD-L1 axis, the effects of metals and metalloids [metal(loid)s] on other clinically relevant immune checkpoints, including CTLA-4, LAG-3, TIM-3, and TIGIT, remain insufficiently characterized. Moreover, most available evidence is derived from preclinical studies, and the clinical significance of metal(loid)-mediated immune checkpoint regulation has yet to be clearly established. In this review, we provide a systematic and comprehensive synthesis of the bidirectional effects of metal(loid)s on the PD-1/PD-L1 axis, the principal molecular signaling networks underlying these effects, and the emerging evidence supporting their potential translational relevance. Although our discussion addresses immune checkpoint regulation more broadly, we acknowledge that the current literature remains predominantly focused on the PD-1/PD-L1 axis. Nevertheless, non-canonical immune checkpoints are increasingly recognized as critical regulators of tumor immune evasion and immunotherapeutic responses, and therefore warrant integration into the broader conceptual framework of metal(loid)-mediated immunomodulation. The selection of metal(loid)s in this review—arsenic, chromium, nickel, cobalt, iron, copper, zinc, and selenium—was based on predefined criteria: the availability of experimental or epidemiological evidence linking these elements to immune checkpoint regulation, particularly the PD-1/PD-L1 axis; their environmental and occupational relevance reflecting common human exposure scenarios; their toxicological and biological importance, encompassing both essential trace elements and toxic metal(loid)s; and the presence of sufficient mechanistic data to support pathway-level synthesis. In contrast, other toxic metal(loid)s such as cadmium, lead, and mercury were excluded from the mechanistic sections due to the lack of direct evidence linking them to immune checkpoint modulation; however, they are discussed in the health risk section to provide a broader view of systemic immunotoxicity and to better reflect real-world mixed-metal exposure. Importantly, evidence from different study designs is critically appraised using a structured framework that distinguishes mechanistic, preclinical, and clinical findings, thereby clarifying their respective evidentiary strengths and translational limitations. In parallel, we comprehensively collate and critically evaluate all available studies on the modulation of other non-canonical immune checkpoints by metal(loid)s, published up to our prespecified literature cutoff. Building on this integrated analysis, we delineate the key unresolved knowledge gaps in this field and highlight priority directions for future mechanistic and translational investigation.

## 2 Methods

We included peer-reviewed original research and review articles published in English that examined the regulation of immune checkpoint expression and function in response to exposure to, or homeostatic disruption of metal(loid)s. Our primary focus was on the PD-1/PD-L1 axis, while also considering other co-inhibitory checkpoints, including CTLA-4, LAG-3, TIGIT, and TIM-3. Eligible studies were required to investigate tumor immunity-related phenotypes and to involve investigations in human subjects, animal models, or mammalian tumor and immune cell systems. We excluded studies that were not directly relevant to metal- or metalloid-mediated modulation of immune checkpoints, as well as non-peer-reviewed articles, non-English publications, studies with critical methodological limitations, and duplicate or redundant reports. All included articles were independently screened by two reviewers, with discrepancies resolved through discussion and consensus with a third senior reviewer.

To more clearly distinguish exploratory from validated findings, we further categorized human evidence according to the degree of clinical validation. Findings were considered exploratory if they were derived from single-center, retrospective, cross-sectional, or small observational studies; if exposure assessment was heterogeneous; or if immune checkpoint outcomes were inferred indirectly. Findings were considered clinically validated only when replicated in independent prospective cohorts, used standardized exposure or biomarker measurements, included clinically relevant endpoints such as response rate, progression-free survival, or overall survival, and demonstrated robustness after adjustment for major confounders. Using these criteria, most currently available human evidence was classified as exploratory rather than validated.

To improve interpretability and translational clarity, the evidence included in this review was systematically classified according to study type and level of biological relevance. Specifically, evidence was grouped into four categories: (i) *in vitro* mechanistic studies (cellular and molecular experiments), (ii) *in vivo* animal studies, (iii) clinical observational studies (retrospective or prospective human data), and (iv) population-level or public health studies. In addition to classification by study type, we further evaluated the translational relevance of each line of evidence based on biological plausibility, consistency across models, and proximity to clinical application. Mechanistic findings derived from *in vitro* or animal models were interpreted as hypothesis-generating, whereas clinical and population-based studies were considered more directly informative for human health implications, albeit with potential confounding factors. Where appropriate, we explicitly indicate the level of evidence supporting each proposed mechanism or association to avoid overinterpretation while highlighting areas requiring further clinical validation.

## 3 Biological characteristics of metal(loid)s relevant to immune modulation

Metal(loid)s vary markedly in their physicochemical properties, biological handling, and toxicokinetics, all of which critically influence their immunological effects. Accordingly, they display distinct patterns of absorption, systemic transport, tissue distribution, and accumulation. Essential trace elements such as iron, copper, zinc, and selenium are tightly regulated by homeostatic networks that involve dedicated transporters, binding proteins, and storage systems (3). Their biological effects are highly dose-dependent and context-specific, with both deficiency and excess capable of disrupting immune function (4). Iron is stored within ferritin in macrophages and undergoes rapid intracellular sequestration during infection, zinc cannot be stored in the body yet undergoes dynamic spatiotemporal redistribution in activated immune cells, and selenium is incorporated into selenoproteins that mediate antioxidant defense and immune modulation (3). By contrast, hazardous metal(loid)s such as arsenic, cadmium, lead, mercury, chromium, and nickel are characterized by high toxicity, environmental persistence, and bioaccumulative potential, which lead to their accumulation in biota and contamination of global food chains (5).These elements tend to accumulate in specific tissues, with arsenic concentrating in the skin and liver and chromium accumulating in lung tissue following inhalation exposure (6). Their toxicity is commonly mediated by oxidative stress, aberrant enzyme-sulfhydryl interactions, and metabolic disruption (5). Differences in metal-induced reactive oxygen species (ROS) levels, antioxidant defense capacity, and organ-specific toxic effects further contribute to variability in immune dysfunction and inflammatory responses (6, 7). These factors should be carefully considered when interpreting experimental findings and extrapolating them to human disease contexts.

## 4 Metal(loid)s’ types and influence on immune checkpoint expression and function

Inhibitory immune checkpoints, comprising receptor–ligand pairs, regulate T-cell activation and tolerance, contributing to self-tolerance while limiting autoimmunity. In cancer, these pathways are often co-opted to promote tumor immune evasion (8). Major checkpoints include PD-1, CTLA-4, LAG-3, TIGIT, TIM-3, and BTLA (B and T lymphocyte attenuator), which are frequently upregulated in exhausted T cells. **Table 1** provides an overview of these key immune checkpoints, including their ligands, biological functions, and current clinical status. Understanding these checkpoints is essential for improving immunotherapy approaches, particularly with ICIs. Engagement of PD-1 with PD-L1 or PD-L2 recruits Src homology region 2 domain-containing phosphatase-1 (SHP-1) and Src homology region 2 domain-containing phosphatase-2 (SHP-2) to Immunoreceptor tyrosine-based switch motif (ITSM)/Immunoreceptor tyrosine-based inhibitory motif (ITIM) motifs in the PD-1 cytoplasmic tail, inhibiting T-cell receptor (TCR) and CD28 signaling and suppressing T-cell function (9). Clinically, ICIs targeting PD-1 (e.g., nivolumab), CTLA-4 (e.g., ipilimumab), and LAG-3 (e.g., relatlimab) have shown efficacy in multiple cancers (10, 11), whereas TIGIT and TIM-3 blockade remain under investigation. Emerging research suggests that metal(loid)s can modulate immune checkpoint expression, often through complex molecular mechanisms. **Table 2** summarizes the effects of various metal(loid)s on immune checkpoint molecules like PD-1 and PD-L1, highlighting the mechanisms involved in these processes. These findings are important for understanding how metal(loid) exposure may influence antitumor immunity, particularly in the tumor immune microenvironment (TIME).

**Table 1:** Overview of major coinhibitory immune checkpoints in cancer: ligands, expression, functions, clinical development and structure.

Molecule	Ligand(s)	Expression pattern	Biological function	Clinical status	Structure (PDB ID/Link)
PD-1	PD-L1, PD-L2 (61)	Activated T cells, B cells, NK cells, macrophages and several subsets of DCs (62)	Maintains immune tolerance, negatively regulates T cell activation, and prevents autoimmune diseases (63)	FDA-approved (e.g., nivolumab, pembrolizumab) for various indications (64, 65)	3BIK (PD-1/PD-L1 Complex; 2.65 Å)
CTLA-4	CD80, CD86 (66)	Constitutively expressed in regulatory T cells (Tregs); inducibly expressed in conventional T cells upon activation (67)	Suppresses T cell activation, maintains immune tolerance, and prevents autoimmunity (68)	FDA-approved (Ipilimumab and tremelimumab) for multiple cancers (69)	1I8L (B7-1/CTLA-4 CO-STIMULATORY COMPLEX; 3.00 Å)
LAG-3	MHC-II, FGL1 (70)	Activated T cells, NK cells, B cells, and plasmacytoid dendritic cells (71)	Promotes T cell exhaustion (71)	FDA-approved (Relatlimab) for unresectable or metastatic melanoma (72)	7TZG (Complex with antibody single-chain variable fragment)
TIGIT	CD155, CD112 (73)	Peripheral memory and regulatory CD4^+^ T cells and NK cells (74)	Inhibits T cell activation and proliferation, suppresses NK cell function, facilitates tumor immune evasion (75)	No FDA approval; Tiragolumab has received Breakthrough Therapy Designation (76), but has not yet obtained formal FDA approval	3UCR (TIGIT IgV domain; 2.63 Å)
TIM-3	Galectin 9, HMGB1, and CEACAM1 (77)	Activated Th1, Th17, and Tc1 cells (78)	Promotes immunosuppression and T cell exhaustion (79)	No FDA approval; In Phase I/II clinical trials (80)	6DHB (TIM-3 with bound Calcium; 1.70Å)
CD47	Thrombospondin-1, SIRPα (81)	All human cells (82)	Provides “Don’t eat me” signal to macrophages (83)	No FDA approval; In Phase Ib/II clinical trials (magrolimab) (84)	2JJS (Complex with SIRP; 1.85 Å)
B7-H3	TLT-2, IL20RA, PLA2R1 (85)	Both cancer and immune cells (86)	Suppresses T cell activation; promotes tumor evasion (87)	No FDA approval; In clinical trials (ifinatamab deruxtecan, DS-7300a) (88)	4I0K (Murine B7-H3 extracellular domain, 2.97 Å)
IDO1	AhR (89)	Tumor cells (90)	Degrades L-tryptophan to kynurenine (Kyn) and enhances immunosuppression (91)	No FDA approval; In clinical trials (BMS-986205, linrodostat) (90)	6O3I (Bound to navoximod; 2.69Å)
BTLA	HVEM (92)	Spleen, lymph nodes, activated T-cells, and resting B-cells (93)	Delivers inhibitory signals or proinflammatory signals (94)	No FDA approval; A phase I/II Study of tifcemalimab (95)	2AW2 (BTLA-HVEM complex; 2.80 Å)
VISTA	VSIG-3, PSGL-1 (96)	Naive CD4^+^ T cells and Treg cells (97)	Suppress T-cell activation (98)	No FDA approval; In Phase I clinical trials (SNS-101) (98)	6OIL (Human VISTA extracellular domain; 1.85 Å)
SIGLEC-15	CD44, CD11b, Muc5B (99)	Myeloid cells and cancer cells (100)	Suppresses T cell function; promotes evasion (100)	No FDA approval; In Phase II clinical trials (NC318) (101)	7ZOZ (Siglec-15 in complex with Fab; 2.10Å)
CD200	CD200R (102)	Almost peripheral cells (103)	Inhibits T-cell immune responses and NK cytotoxic activity (104)	No FDA approval; In Phase I clinical trials (samalizumab) (105)	4BFI (The extracellular portions of mouse CD200R and mouse CD200; 3.22 Å)
CD160	HVEM (106)	NK cells and CD8 T -lymphocytes (107)	Suppresses T cell cytotoxicity (108)	No FDA approval	6NG9 (Human; 1.95 Å)

AhR, aryl hydrocarbon receptor; BTLA, B and T lymphocyte attenuator; DCs, dendritic cells; FDA, U.S. Food and Drug Administration; FGL1, fibrinogen-like protein 1; HMGB1, high-mobility group box 1; HVEM, herpesvirus entry mediator; IDO1, indoleamine 2,3-dioxygenase 1; Kyn, kynurenine; MHC-II, major histocompatibility complex class II; PDB, Protein Data Bank; PSGL-1, P-selectin glycoprotein ligand 1; SIGLEC-15, sialic acid-binding immunoglobulin-like lectin 15; SIRPα, signal regulatory protein alpha; TIGIT, T-cell immunoreceptor with immunoglobulin and immunoreceptor tyrosine-based inhibitory motif domains; TIM-3, T-cell immunoglobulin and mucin-domain-containing 3; Tregs, regulatory T cells; VISTA, V-domain immunoglobulin suppressor of T-cell activation; VSIG-3, V-set and immunoglobulin domain containing 3.

**Table 2:** Mechanisms of metal(loid)s-induced modulation of PD-1/PD-L1 axis.

Mechanism	Compound form	Effect on immune checkpoints	Regulatory pathways	Model	Experimental dose range	ED	Reference
TIME Remodeling & Immune Cell Regulation	SeNPs; Methylseleninic acid	↓ PD-L1	Activate Selenoproteins→M2→M1 TAMs polarization + IL-15/NKG2D axis↑+ Th1 cytokine (IFN-γ)↑→PD-1 (on CIK cells)↓+ PD-L1 (on tumor cells)↓→PD-1/PD-L1↓	*In vitro*; Animal	4 μM SeNPs; 5 μM Methylseleninic acid	24 h	(25, 109, 110)
	NiSO_4_; Ni as contact allergen	↑ PD-L1	Activate LCs/MoLCs→PD-L1↑→ enhance PD-L1/PD-1 interaction→ suppress proinflammatory cytokines	*In vitro*	100–400 μM NiSO_4_	24–72 h	(16)
	High dietary Zn; ZnSO_4_·7H_2_O; Zn-deficient diet	↑ PD-1 (high Zn);↓ PD-1 (Zn deficiency)	Inhibit SIRT1→FOXO1↓ deacetylation→FOXO1–FOXP 3 binding↑→↑FOXP3^+^ Treg expansion (TGF-β/NFAT1↑)→Th1 responses↓(IFN-γ, TNF-α↓)→T-cell exhaustion→PD-1↑	*In vitro*; Animal	15 μM, 50 μM ZnSO_4_·7H_2_O	48–72 h	(24)
ROS-Mediated PD-1/PD-L1 Regulation	Ferlixit (Fe³^+^-containing); Iron in TIME	↑ PD-L1	Induce ROS production→promote c-Myc nuclear translocation→ PD-L1↑	*In vitro*	250 μM	24 h	(18)
	ATO	↑ PD-L1	Induce ROS→cause DNA damage→ trigger Cyclin D1 degradation→ inhibit CDK4/6→downregulate Cul3 →stabilize PD-L1→PD-L1↑	*In vitro*	10 μM	48 h	(13)
Hypoxia-Induced PD-L1 Regulation	CoCl_2_, hypoxia mimetic	↑ PD-L1	Activate HIF-1α→bind to HRE-4 in PD-L1 promoter→PD-L1↑	*In vitro*	30 mg/kg, 60 mg/kg	6 h	(17)
JAK/STAT3-Mediated Signaling	Sodium arsenite	↑ PD-L1	Activate STAT3 phosphorylation (Tyr705)→PD-L1↑	Animal	500 ppb	1 year	(12)
	CuCl_2_	↑ PD-L1	Activate EGFR/STAT3 signaling→PD-L1↑	*In vitro*	20–200 μmol/L	24 h	(22)
	Copper chelators	↓ PD-L1	Inhibit EGFR–STAT3 phosphorylation →PD-L1↓	*In vitro*	2 mmol/L	24 h	(22)
	Cr(VI), K_2_Cr_2_O_7_, CaCrO_4_	↑ PD-L1	Upregulate lncRNA *ABHD11-AS1*→SART3 binding→reduce cytoplasmic USP15→increase TRAF3 ubiquitination and proteasomal degradation→noncanonical NF-κB (RelB/p52)↑→IL-6↑→ JAK/STAT3↑→PD-L1 stability↑→ PD-L1↑	*In vitro*; Animal	100 μg/week	16 weeks	(15)
Tumor Suppressor & Protein Homeostasis	As_4_S_4_	↓ PD-L1	Activate p53/*miR-34a-5p* axis→ PD-L1↓	*In vitro*; *In vivo*; Animal	0.5–3.0 μM 1 mg/kg qd	48 h 3 weeks	(14)
	Zinc L-carnosine	↑ PD-L1	Inhibit miR-570→PD-L1↑	*In vitro*; Animal	10 mM	24 h	(23)
	ATO	↑ PD-L1	Downregulate Cul3→reduce ubiquitination→stabilize PD-L1→ PD-L1↑	In animal	2 mg/kg	3 weeks	(13)
	Copper chelators	↓ PD-L1	Promote ubiquitin-mediated PD-L1 degradation→PD-L1↓	*In vitro*	20 μg/mL	24 h	(22)

ATO, arsenic trioxide; CIK, cytokine-induced killer; CUL3, Cullin 3; DC, dextran-catechin; EGFR, epidermal growth factor receptor; FOXP3, forkhead box P3; HIF-1α, hypoxia-inducible factor 1 alpha; HRE-4, hypoxia response element 4; IL-6, interleukin-6; LC, Langerhans cell; lncRNA, long non-coding RNA; miR, microRNA; MoLC, monocyte-derived Langerhans cell; NF-κB, nuclear factor kappa B; NKG2D, natural killer group 2D; qd, once daily; ROS, reactive oxygen species; SIRT1, sirtuin 1; STAT3, signal transducer and activator of transcription 3; TAM, tumor-associated macrophage; TEPA, tetraethylenepentamine; TIME, tumor immune microenvironment; TRAF3, TNF receptor-associated factor 3; USP15, ubiquitin-specific peptidase 15. ↑ indicates upregulation, activation, enhancement, or increased expression/activity; ↓ indicates downregulation, inhibition, suppression, or decreased expression/activity.

### 4.1 Arsenic

Evidence from *in vitro* and *in vivo* studies suggests that arsenic exerts form-dependent and bidirectional effects on the PD-1/PD-L1 axis. Inorganic arsenic, specifically sodium arsenite, promotes lung tumor immune evasion by activating signal transducer and activator of transcription 3 (STAT3) at tyrosine 705 (Tyr705) specifically in alveolar epithelial type 2 (AT2) cells following 12 months of chronic exposure to 500 ppb in drinking water, thereby upregulating PD-L1 expression on AT2 cells and impairing CD8^+^ T-cell-mediated antitumor immunity (12). Similarly, arsenic trioxide (ATO) drives the time-dependent upregulation of PD-L1 via the cyclin D1-cyclin-dependent kinase 4/6 (CDK4/6) signaling cascade. *In vitro*, exposing esophageal squamous cell carcinoma (ESCC) and oral squamous cell carcinoma (OSCC) cell lines to 10 μM ATO for 48 hours significantly increases PD-L1 expression. Furthermore, *in vivo* intraperitoneal administration of ATO (2 mg/kg, every other day for 3 weeks) mirrors these findings, thereby sensitizing tumors to PD-L1 blockade therapy (13). In contrast, tetraarsenic tetrasulfide (As_4_S_4_) downregulates PD-L1 (0.5–3.0 μM, 48 h *in vitro*; 1 mg/kg qd × 3 weeks *in vivo*), enhances cisplatin-induced apoptosis in chemoresistant non-small-cell lung cancer (NSCLC) cells, and synergizes with cisplatin, indicating distinct immunological effects of different arsenic species (14).

### 4.2 Chromium

Preclinical studies indicate that chromium, particularly hexavalent chromium, may exert pro-carcinogenic and immunosuppressive effects. Experimental evidence demonstrates that hexavalent chromium [Cr(VI)] increases PD-L1 expression through long noncoding RNA (lncRNA) ABHD11-AS1-mediated nuclear translocation of ubiquitin-specific peptidase 15 (USP15), which promotes tumor necrosis factor (TNF) receptor-associated factor 3 (TRAF3) ubiquitination and proteasomal degradation, activates non-canonical nuclear factor kappa B (NF-κB) and downstream interleukin-6 (IL-6)-Janus kinase (JAK)/STAT3 signaling, and stabilizes PD-L1 protein following 16 weeks of chronic oropharyngeal exposure to 100 μg calcium chromate (CaCrO_4_) once weekly in A/J mice, or in chronically potassium dichromate (K_2_Cr_2_O_7_)-transformed human bronchial epithelial BEAS-2B cells (15). These findings suggest that Cr(VI) promotes tumor immune evasion through coordinated transcriptional and post-translational regulation of PD-L1.

### 4.3 Nickel

Evidence from *in vitro* studies suggests that nickel may enhance PD-L1-dependent immunosuppressive signaling, although the currently available evidence remains limited. Nickel sulfate (NiSO_4_) induces PD-L1 expression on human Langerhans cells (LCs) in a dose-dependent manner (100–400 μM *in vitro*, 5% topical application *in vivo*), with maximal upregulation observed at 24 hours in monocyte-derived LCs (MoLCs) and at 72 hours in epidermal LCs of allergic contact dermatitis patients, thereby reducing IL-22 production by Th cells and tumor necrosis factor-alpha (TNF-α) and attenuating immune responses through the PD-1/PD-L1 axis (16). Although direct evidence in tumor models is still scarce, these observations support a potential role for Ni in reinforcing inhibitory immune signaling.

### 4.4 Cobalt

Experimental studies suggest that cobalt, commonly modeled by cobalt chloride exposure, may promote an immunosuppressive microenvironment by mimicking hypoxia. Mechanistically, Cobalt chloride (CoCl_2_) induces hypoxia-inducible factor 1 alpha (HIF-1α)-dependent PD-L1 transcription in tumor and myeloid cells in a dose-dependent manner (30–60 mg/kg intraperitoneal injection *in vivo*, 0.1% pO_2_ hypoxic culture *in vitro*), with significant PD-L1 upregulation observed at 6 hours in splenic myeloid-derived suppressor cells (MDSCs) of tumor-bearing mice and at 24–72 hours in cultured MDSCs, macrophages, dendritic cells, and multiple tumor cell lines, thereby enhancing MDSC-mediated immunosuppression; importantly, PD-L1 blockade can reverse these effects by downregulating MDSC-derived IL-6 and IL-10 production (17). This suggests that cobalt-driven hypoxic signaling may contribute to tumor immune escape and may represent a therapeutically targetable pathway.

### 4.5 Iron

Preclinical studies suggest that iron, as an essential trace element, may exert bidirectional effects on antitumor immunity depending on systemic status and cellular context. Ferric iron (Fe³^+^) induces ROS production, activates c-Myc nuclear translocation and upregulates PD-L1 transcription in lung adenocarcinoma (LUAD) cells at a concentration of 250 μM after 24 hours of treatment, and this effect is completely abrogated by the antioxidant trolox (200 μM for 6 hours); importantly, iron-induced PD-L1 overexpression significantly suppresses T-cell function by reducing interferon gamma (IFN-γ) release in a 3-hour co-culture system, and c-Myc transient knockdown (48 hours) fully reverses the iron-mediated PD-L1 upregulation (18).Conversely, appropriate iron supplementation can enhance T-cell activity and improve the efficacy of PD-1 blockade, suggesting that the clinical impact of iron is closely related to systemic iron levels and tumor context (19, 20).

### 4.6 Copper

Experimental studies indicate that copper may promote PD-L1-mediated immune evasion in tumors. Increased copper availability (as Copper(II) chloride (CuCl_2_), 1 mmol/L for up to 8 hours *in vitro*) enhances (epidermal growth factor receptor) EGFR (Y1068) and STAT3 (Y705) phosphorylation in neuroblastoma and glioblastoma cells, and copper supplementation dose-dependently (20–200 μmol/L for 24 hours *in vitro*) drives PD-L1 mRNA and protein expression; conversely, copper chelators (dextran-catechin, DC, 20 μg/mL for 24 hours *in vitro*; tetraethylenepentamine, TEPA, 2 mmol/L for 24 hours *in vitro*) inhibit EGFR/STAT3 phosphorylation, promote ubiquitin-mediated proteasomal degradation of PD-L1 (reducing its half-life from 23.28 hours to 13.76 hours), and *in vivo*, oral TEPA (400 mg/kg/day for 72 hours to 1 week) or intravenous DC (300 mg/mL single dose for 24–48 hours) significantly downregulate tumor PD-L1 expression in *Th-MYCN* neuroblastoma mice (21, 22). These findings suggest that copper-lowering strategies may enhance the efficacy of ICIs.

### 4.7 Zinc

Clinical and experimental studies suggest that zinc exhibits context-dependent effects on the PD-1/PD-L1 axis. Zinc L-carnosine (ZnC) suppresses *miR-570* expression and thereby upregulates PD-L1 mRNA and protein levels in colorectal cancer (CRC) cells at a concentration of 10 mM after 24 hours of *in vitro* treatment, and daily oral gavage of ZnC at 100 mg/kg for 21 days in C57BL/6 mice bearing *MC38* CRC xenografts significantly enhances the antitumor efficacy of anti-PD-1 therapy (administered intraperitoneally at 200 μg every 3 days starting on day 8), which is associated with increased CD8^+^ T-cell infiltration and reduced MDSCs in the tumor microenvironment (23). However, excessive dietary zinc (as zinc sulfate (ZnSO_4_·7H_2_O), 80 mg/kg/day administered orally from day 2 post-tumor implantation until endpoint ~21 days) promotes the expansion of forkhead box P3 (FOXP3^+^) regulatory T cells (Tregs) and upregulates PD-1 expression on CD4^+^, CD8^+^ and γδ T cells in the *B16F10* melanoma tumor microenvironment; *in vitro*, zinc supplementation dose-dependently (15–50 μM for 48–72 hours) increases PD-1 surface expression on activated T cells, enhances naive CD4^+^ T cell differentiation into Tregs and suppresses T helper (Th) cell differentiation, thereby collectively inhibiting Th1-type antitumor responses and driving T-cell exhaustion (24). Collectively, these findings indicate that the immunological effects of zinc are highly dependent on dose, formulation, and microenvironmental context.

### 4.8 Selenium

Most current evidence regarding selenium-mediated immune modulation is derived from experimental models. In particular, selenium nanoparticles (SeNPs) and methylseleninic acid can downregulate PD-1 on effector immune cells and PD-L1 on tumor cells, thereby alleviating immune exhaustion and enhancing antitumor responses (25). These findings support the potential of selenium as an adjuvant for improving antitumor immunity and potentiating immunotherapy.

### 4.9 Real-world mixed metal(loid) exposures and immune checkpoint regulation

Unlike the single-metal exposure designs used in most preclinical studies, real-world environmental and occupational exposures almost always involve complex mixtures of metal(loid)s. For example, drinking water typically contains concurrent arsenic, cadmium, and lead (26), while occupational exposure in electroplating workers often involves chromium, nickel, and cobalt mixtures.

However, current research on mixed metal(loid) exposures remains extremely limited. Most studies have focused on general immunotoxicity rather than immune checkpoint regulation, and the interaction networks between different metal(loid)s in the tumor microenvironment remain poorly understood.

### 4.10 Current state of research on non-PD-1/PD-L1 immune checkpoints

In contrast to the relatively extensive evidence involving the PD-1/PD-L1 axis, the potential effects of metal(loid)s on other immune checkpoints remain poorly characterized. Nevertheless, in addition to PD-1/PD-L1 signaling, several biological processes influenced by metal(loid)s, including oxidative stress, inflammatory signaling, hypoxia-associated responses, and epigenetic remodeling, have also been implicated in the regulation of other inhibitory immune checkpoints, such as CTLA-4, TIM-3, LAG-3, and TIGIT (27–30). Although direct experimental evidence remains limited, these observations suggest that metal(loid)-mediated immunomodulation may extend beyond the PD-1/PD-L1 axis. Considering the growing clinical importance of non-canonical immune checkpoints in next-generation immunotherapy, further investigation into their interactions with environmental metal(loid) exposure is warranted.

Overall, metal(loid)s exert diverse and context-dependent modulatory effects on immune checkpoints, predominantly via PD-1/PD-L1 signaling, with significant implications for tumor immune evasion, antitumor immunity, and therapeutic responses to ICIs.

## 5 Mechanisms of metal(loid)-induced modulation of immune checkpoints

**Figure 1:**
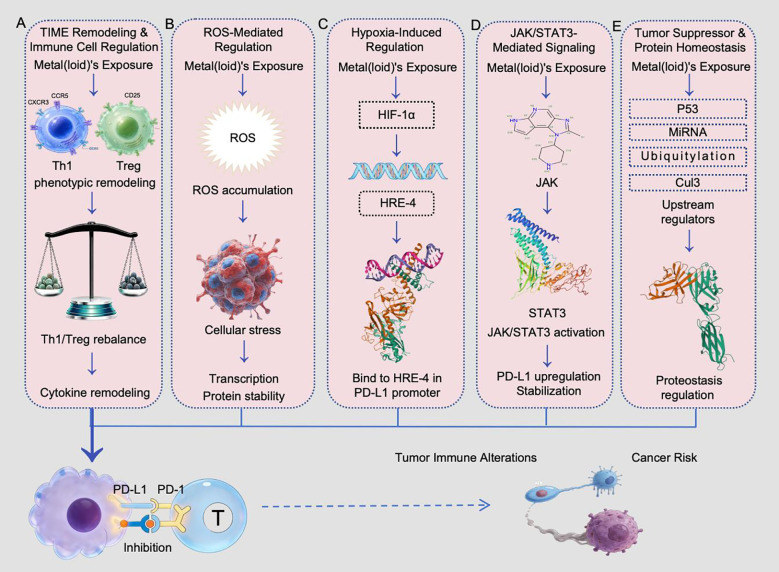
Potential mechanisms underlying metal(loid) exposure-induced PD-L1 regulation and their implications for tumor immune alterations and cancer risk; **(A)** Metal(loid) exposure remodels the tumor immune microenvironment (TIME) by altering immune cell phenotypes, including the Th1/Treg balance, and by reshaping cytokine profiles; **(B)** Metal(loid) exposure induces ROS accumulation and tumor cell stress responses, leading to transcriptional regulation and/or altered protein stability of PD-L1; **(C)** Metal(loid) exposure activates hypoxia-related signaling, including HIF-1α, which binds to the HRE-4 site in the PD-L1 promoter and promotes PD-L1 expression; **(D)** Metal(loid) exposure activates the JAK/STAT3 signaling pathway, resulting in PD-L1 upregulation and/or stabilization; **(E)** Metal(loid) exposure may also regulate PD-L1 through tumor suppressor- and protein homeostasis-related pathways, including p53, miRNAs, Cul3, and ubiquitylation-associated mechanisms. Collectively, these pathways converge on PD-L1/PD-1-mediated immunosuppressive signaling, thereby contributing to tumor immune alterations and cancer risk modification. TIME, tumor immune microenvironment; Th1, T helper 1; Treg, regulatory T cell; ROS, reactive oxygen species; HIF-1α, hypoxia-inducible factor 1 alpha; HRE-4, hypoxia response element 4; PD-L1, programmed death-ligand 1; PD-1, programmed cell death protein 1; miRNA, microRNA; Cul3, Cullin 3.

Building on the mechanistic evidence discussed above, metal(loid)-mediated immune checkpoint regulation can be broadly classified into several interconnected signaling networks. These mechanisms encompass remodeling of the TIME, ROS-dependent transcriptional and post-translational regulation of PD-L1, hypoxia/HIF-1α-driven signaling, activation of inflammatory JAK/STAT3 pathways, and disruption of tumor suppressor signaling and protein homeostasis. Although presented individually for conceptual clarity, these pathways are highly integrated and collectively contribute to PD-1/PD-L1-mediated immune evasion and suppression of antitumor immunity. The major mechanistic pathways discussed in this section are summarized schematically in **Figure 1** and systematically integrated with the supporting evidence presented in **Table 2**.

### 5.1 TIME remodeling and immune cell function regulation pathway

Metal(loid)s can modulate the TIME by reprogramming immune cell functions, thereby modulating PD-1/PD-L1 signaling and shaping antitumor immune responses (**Figure 1A**). Specifically, SeNPs activate selenoproteins, which in turn drive the polarization of tumor-associated macrophages (TAMs) from an M2 to an M1 phenotype. This polarization enhances IL-15/natural killer group 2D (NKG2D) signaling and promotes Th1-type cytokine IFN-γ secretion, ultimately resulting in decreased PD-1 expression on cytokine-induced killer cells and reduced PD-L1 expression on tumor cells (25). By contrast, nickel activates LCs and MoLCs, leading to upregulation of PD-L1. This upregulation suppresses proinflammatory cytokine release from T helper (Th) cells (16) and may contribute to the establishment of an immunosuppressive TIME. Furthermore, zinc exhibits dose-dependent biphasic effects on PD-1: elevated zinc levels inhibit Sirtuin 1 (SIRT1), enhance FOXO1–FOXP3 interaction, and promote Treg expansion via transforming growth factor-β (TGF-β)/nuclear factor of activated T cells 1 (NFAT1) signaling, thereby reducing Th1 cytokine secretion (IFN-γ and TNF-α) and increasing PD-1 expression. In contrast, zinc deficiency produces the opposite effects. Clinical observations indicate that high zinc intake reduces the efficacy of anti-PD-1 ICIs, while dietary zinc deficiency or tissue zinc chelation significantly enhances the antitumor response of ICIs (24). Collectively, these findings demonstrate that metal(loid)-mediated TIME remodeling integrates immune cell reprogramming with PD-1/PD-L1 regulation, emphasizing the potential of targeting cell-specific pathways to optimize immunotherapy in populations with differential metal(loid) exposure.

### 5.2 ROS-mediated immune checkpoint regulation

Metal(loid)s also regulate PD-L1 expression through ROS-dependent pathways, which modulate T cell activity and promote immunosuppression within the tumor microenvironment (**Figure 1B**). For example, Fe³^+^ induces mitochondrial ROS overproduction, which activates c-Myc and facilitates its nuclear translocation. Once in the nucleus, c-Myc transcriptionally upregulates PD-L1, thereby suppressing T cell effector functions, including IFN-γ secretion (18). Similarly, ATO triggers ROS-mediated DNA damage, leading to cyclin D1 phosphorylation and degradation, downregulation of CUL3 (Cullin 3) E3 ligase activity, and stabilization of PD-L1 protein (13). Notably, antioxidant treatment may reverse these effects, confirming ROS as a central mediator of metal(loid)-driven immune checkpoint regulation. These findings highlight ROS signaling as a central regulatory axis and suggest that co-targeting ROS and immune checkpoints may enhance antitumor efficacy, pending formal validation in future studies.

### 5.3 Hypoxia signaling axis-mediated PD-L1 regulation

Metal(loid)s can upregulate PD-L1 expression via hypoxia signaling pathways, thereby shaping an immunosuppressive tumor microenvironment (**Figure 1C**). Specifically, CoCl_2_ stabilizes HIF-1α, which binds to the hypoxia response element 4 (HRE-4) element within the PD-L1 promoter. This binding selectively increases PD-L1 expression on MDSCs, macrophages, dendritic cells, and tumor cells. As a result, T cell proliferation is inhibited, IFN-γ secretion is reduced, and the production of immunosuppressive cytokines IL-6 and IL-10 is elevated, collectively facilitating immune evasion (17). Notably, HIF-1α also transcriptionally upregulates multiple potassium channels (including voltage-gated potassium, big conductance KCa, and potassium inwardly-rectifying channel subfamily J member) under hypoxic conditions, which in turn amplify hypoxia-induced tumor cell migration, and stemness acquisition, forming a positive feedback loop between hypoxia signaling and ion channel dysregulation (31). HIF-1α-mediated hypoxia signaling constitutes a core pathway for metal(loid)-induced PD-L1 upregulation, providing a mechanistic rationale for combined targeting of HIF-1α and PD-1/PD-L1 to enhance antitumor immunity.

### 5.4 JAK/STAT3 core immune signal transduction pathway

Metal(loid)s further modulate PD-L1 expression through the JAK/STAT3 core immune signaling axis, thereby reshaping the immunosuppressive tumor microenvironment and promoting tumor immune escape (**Figure 1D**). For instance, sodium arsenite induces phosphorylation of STAT3 at Tyr705, which upregulates PD-L1 in AT2 cells. Deletion of STAT3 abolishes this effect and prevents arsenic-induced lung tumorigenesis (12). Copper(II) chloride (CuCl_2_) activates the EGFR–STAT3 cascade, resulting in elevated PD-L1 mRNA and protein levels, whereas copper chelators reverse these effects via ubiquitin-mediated degradation (22). In addition, Cr(VI) activates JAK/STAT3 through the lncRNA ABHD11-AS1→USP15→TRAF3→NF-κB→IL-6 signaling pathway, stabilizing PD-L1 protein in lung tissues (15). These findings underscore the JAK/STAT3 pathway as a conserved node in metal(loid)-mediated PD-L1 regulation, highlighting STAT3 as a potential therapeutic target for reversing metal(loid)-associated immune suppression.

### 5.5 Tumor suppressor signaling and protein homeostasis regulation pathway

Metal(loid)s regulate PD-L1 via tumor suppressor signaling and protein homeostasis mechanisms, which include microRNA (miRNA)-mediated post-transcriptional regulation and CUL3-dependent ubiquitination (**Figure 1E**). For example, As_4_S_4_ downregulates PD-L1 through activation of the p53/miR-34a-5p (microRNA-34a-5p) axis, reversing cisplatin resistance in NSCLC (14). ZnC increases PD-L1 expression by inhibiting *miR-570*-mediated post-transcriptional silencing (23). Similarly, miR-424-5p negatively regulates protein kinase B (PKB) to suppress PKB/mammalian target of rapamycin pathway activation (32) and subsequent PD-L1 expression. Furthermore, ATO and copper chelators modulate PD-L1 protein stability via CUL3/ubiquitin pathways, either stabilizing or promoting PD-L1 degradation, respectively (13, 22). These mechanisms demonstrate that tumor suppressor and protein homeostasis pathways provide a bidirectional regulatory mechanism for PD-L1, representing promising targets to optimize ICI therapy.

## 6 Tumor type-specific heterogeneity in metal(loid)-mediated immune checkpoint regulation

The immunomodulatory effects of metal(loid)s are highly context-dependent and may vary substantially across tumor types. Different tumor types exhibit distinct patterns of metal homeostasis, tissue accumulation patterns, TIME characteristics, and regulatory mechanisms governing immune checkpoint expression. For instance, lung tissues may be particularly vulnerable to inhaled metal exposure, especially chromium and nickel compounds. Experimental evidence indicates that nickel exposure can induce PD-L1 expression in LCs and modulate T-cell cytokine responses through PD-1/PD-L1 signaling, suggesting that epithelial–immune cell interactions may contribute to local immune checkpoint dysregulation in metal-exposed tissues (16). Hepatocellular carcinoma is closely linked to systemic and hepatic metal metabolism, particularly iron and copper homeostasis, given the central role of the liver in metal storage, transport, detoxification, and metabolic regulation (33, 34). In CRC, metal(loid)s may further influence immune responses and immune checkpoint signaling through interactions with the gut microbiota, which is increasingly recognized as an important regulator of colorectal tumor immunity and immunotherapy responsiveness (35–37). Melanoma and breast cancer may also exhibit differential sensitivities to metal(loid)-mediated immunomodulation because of their distinct immune infiltration patterns, tumor microenvironment characteristics, and PD-L1 regulatory networks (38, 39). These variations may ultimately influence responsiveness to ICIs. Therefore, future studies should incorporate tumor type-specific analyses when evaluating metal(loid)-associated immune effects, rather than assuming uniform mechanisms across malignancies. Such stratification will be essential for translating mechanistic findings into precision immuno-oncology.

## 7 Health risks and effects of metal(loid)s exposure on immune response and health

Metal(loid)s have been reported to modulate immune checkpoint signaling, potentially through dysregulated PD-1/PD-L1 signaling, which may contribute to an immunosuppressive milieu and reduced antitumor immune responses. PD-1 expression on CD8^+^ T cells has been observed to increase following TCR activation and can persist during chronic stimulation (40). PD-1/PD-L1 engagement inhibits TCR signaling and CD28 co-stimulation, thereby attenuating CD8^+^ T-cell activation, proliferation, and effector functions (41). Copper dyshomeostasis can upregulate PD-L1 expression via the interleukin-6 (IL-6)/JAK/STAT3 signaling pathway, which has been associated with T-cell exhaustion (42). FOXP3^+^ CD4^+^ Tregs also contribute to immunosuppression. Tregs maintain immune homeostasis through mechanisms such as secretion of immunosuppressive cytokines, such as interleukin-10 (IL-10) and TGF-β, and direct cell-cell contact (43). Within the tumor microenvironment, increased Treg abundance is associated with reduced antitumor immunity and suppression of CD8^+^ T-cell responses (44). Iron accumulation in TAMs can promote proinflammatory M1-like phenotypes with antitumor activity, depending on iron metabolic status, while the core iron metabolic feature of M2-like TAMs is an “iron-recycling” phenotype characterized by higher ferroportin expression and lower ferritin levels, enabling iron export rather than retention (45). ROS generated by immune cells have dual roles: moderate levels can support T-cell activation, whereas elevated excessive levels may contribute to T-cell dysfunction and tumor progression (46). Natural killer (NK) cell function can be affected by metal(loid) exposure, with cadmium being associated with changes in NK-cell cytotoxic activity and lead (Pb) exposure being associated with alterations in NK-cell populations (47). Collectively, these metal(loid)-induced alterations may contribute to resistance to ICIs or reduced therapeutic efficacy and may increase the risk of tumorigenesis.

Although the primary mechanistic focus of this review is on eight selected metal(loid)s, additional toxic metals such as cadmium, lead, and mercury are included in this section due to their well-established systemic toxicity and immunomodulatory effects. These elements are highly relevant in real-world exposure scenarios, where mixed-metal exposure is the norm rather than the exception, and may indirectly influence immune checkpoint pathways through broader immune dysregulation. Cadmium, lead, and mercury exhibit pronounced organ-specific toxicities that can secondarily impact immune function. Cadmium preferentially accumulates in the kidney, where it induces oxidative stress, mitochondrial dysfunction, and oxidative damage to cellular macromolecules (48). Lead is well known for its neurotoxicity and hematological effects, skewing Th cell differentiation and impairing the development and function of immune cells (49). Mercury, particularly in its organic forms, exerts potent neurotoxic and immunotoxic effects and can alter cytokine signaling and immune cell activation (50). These organ-specific toxicities contribute to systemic immune imbalance and may create conditions that favor immunosuppression. Although the systemic toxicity of metal(loid)s has traditionally been described in terms of organ-specific effects, its relevance to cancer lies in the capacity to reshape tumor immunity and immune checkpoint regulation. Chronic exposure to metal(loid)s can induce oxidative stress and persistent inflammation, activating signaling pathways such as NF-κB, STAT3, and HIF-1α, which are known to regulate PD-L1 expression and promote an immunosuppressive TIME. These alterations may impair cytotoxic T-cell function, drive T-cell exhaustion, and facilitate tumor immune evasion, thereby potentially influencing the efficacy of ICIs. In addition, both essential and toxic metal(loid)s may disrupt immune homeostasis through mechanisms including redox imbalance, epigenetic modification, and altered cytokine signaling. While organ-specific toxicities such as respiratory, hepatic, renal, neurological, and endocrine dysfunction may further modulate local and systemic immune responses, their immunological consequences may ultimately converge on common pathways that regulate tumor progression and therapeutic response. Importantly, current evidence is largely derived from preclinical studies, with limited clinical validation. Overall, metal(loid)-induced toxicity should be understood not only as a systemic health risk but also as a critical modulator of tumor immunity and immunotherapy outcomes.

## 8 Integration of multi-level evidence and translational limitations

A major challenge in this field is the integration of evidence across multiple biological and methodological scales. The current literature encompasses *in vitro* mechanistic studies, animal models, clinical observations, and population-based research; however, these evidence types differ substantially in their strengths, limitations, and translational implications. Mechanistic studies provide critical insights into molecular pathways, such as PD-L1 regulation and oxidative stress signaling, but often rely on simplified systems that may not fully capture tumor complexity. Animal models offer improved physiological relevance but may still differ from human immune responses. In contrast, clinical and epidemiological studies provide direct human data but are frequently limited by confounding, heterogeneity in exposure assessment, and lack of mechanistic resolution. Importantly, the majority of evidence linking metal(loid)s to immune checkpoint regulation remains at the preclinical stage. While certain elements, such as zinc, have shown potential clinical associations with ICI outcomes, these findings require validation in prospective and mechanistically informed studies. Similarly, selenium-related evidence is largely based on nanoparticle or supplementation models, which may not directly translate to endogenous selenium biology. Therefore, caution is warranted when extrapolating mechanistic findings to clinical applications, and future research should prioritize integrative approaches that bridge experimental and clinical evidence.

## 9 Knowledge gaps and future prospects

### 9.1 Conceptual gaps and interindividual variability

Several important conceptual gaps remain in understanding how metal(loid)s influence immune checkpoint regulation. A major limitation is the insufficient integration of real-world exposure complexity, particularly mixed-metal(loid) exposure scenarios, which better reflect environmental and occupational conditions but remain underexplored in current studies.

Another important gap is interindividual variability in susceptibility to metal(loid)s. Available evidence suggests that age and sex are associated with internal dose, also referred to as body burden, and that genetic factors are key determinants of toxic effects associated with metal(loid) exposure (51). Children may be more susceptible than adults, partly due to the incomplete development of biotransformation and excretory pathways (52). In addition, sex-specific associations between metal(loid) exposure and immune-related outcomes have been reported, with effect estimates differing between females and males (53). Genetic variants, such as single-nucleotide polymorphisms, may contribute to interindividual differences in toxicokinetics, including absorption, distribution, metabolism, excretion, internal dose, and antioxidant defense capacity (51).

These differences may also reflect variation in exposure sources, lifestyle factors such as diet and tobacco smoking, and sociodemographic characteristics, as well as age-related differences in biotransformation and excretory capacity (54).

However, these modifying factors have not been systematically evaluated in the context of immune checkpoint modulation or combined metal(loid) exposures. A major limitation of the current literature is the overwhelming predominance of studies focused on the PD-1/PD-L1 axis, whereas the potential effects of metal(loid)s on other immune checkpoints, including CTLA-4, TIM-3, LAG-3, and TIGIT, remain largely unexplored. Future research should therefore move beyond the current PD-1/PD-L1-centric paradigm and systematically investigate whether metal(loid)s exert broader immunomodulatory effects across multiple immune checkpoint pathways.

### 9.2 Context-dependent effects and unresolved mechanistic questions

Current evidence suggests that the immunological effects of metal(loid)s are highly context dependent, yet the determinants of these differential effects remain incompletely understood. For example, clinical observational studies have reported that elevated serum zinc levels after 3 weeks of ICI treatment are significantly associated with improved overall survival in patients with advanced cancer, suggesting its potential role as a noninvasive prognostic biomarker (55). In contrast, mechanistic studies have shown that high zinc levels can promote the expansion of FOXP3^+^ Tregs, upregulate PD-1 expression on T cells, suppress Th1-type antitumor immune responses, and potentially reduce the efficacy of anti-PD-1 therapies (24).

These findings are not inherently contradictory but reflect differences in dose dependency, spatial distribution, timing of measurement, and baseline patient characteristics. Specifically, increases in serum zinc associated with improved outcomes may reflect restoration of physiological homeostasis following correction of deficiency, whereas immunosuppressive effects are more often linked to supraphysiological supplementation or abnormal accumulation within the tumor microenvironment (24, 55).

Similarly, arsenic compounds exhibit bidirectional effects on PD-L1 regulation that depend on chemical form, exposure dose, cellular genotype, and molecular pathway specificity. Arsenic sulfide can suppress PD-L1 expression through activation of the p53/*miR-34a-5p* axis in p53-wild-type tumors, whereas arsenic trioxide can induce PD-L1 upregulation through ROS-mediated DNA damage and inhibition of ubiquitin-dependent degradation pathways (13, 14).

Iron also demonstrates dual effects, with local iron overload promoting tumor immune evasion through ROS-mediated signaling, while physiological supplementation may enhance T-cell-mediated antitumor responses and improve immunotherapy efficacy (18–20).

These context-dependent effects are driven by multiple factors, including dose thresholds, spatial distribution between systemic circulation and the TIME, chemical speciation, and cell type-specific signaling pathways.

Despite these insights, key questions remain unresolved, including dose-response relationships within clinically relevant exposure ranges, spatial heterogeneity, and the net effects of mixed-metal(loid) exposure. Metal(loid)-related health risks might be amplified by mixed exposures, which are hypothesized to produce synergistic toxicity that could surpass individual effects. Preliminary observations suggest combined arsenic, chromium, and nickel exposure may elevate lung cancer risk, potentially through synergistic PD-L1 upregulation and impaired antitumor immunity. Likewise, cadmium–lead co-exposure could worsen immune dysfunction by possibly inhibiting NK cell activity and increasing T-cell PD-1 expression, though definitive evidence is lacking.

### 9.3 Methodological limitations and research needs

Methodological challenges further limit the comparability and interpretability of existing studies. A major issue is the lack of standardized exposure assessment, as metal(loid) levels are measured using different biological matrices, analytical methods, and time points across studies. Establishing harmonized protocols for exposure quantification, including both toxic and essential elements such as lead and mercury, represents a critical and immediately actionable priority.

Another limitation is the predominance of cross-sectional and reductionist study designs. Most studies evaluate single metal(loid)s in isolation, whereas real-world exposures involve complex mixtures with potential synergistic or antagonistic effects. In addition, longitudinal data capturing dynamic changes in exposure, immune status, and treatment response remain scarce.

Emerging multi-omics approaches, including genomics, transcriptomics, proteomics, metabolomics, and metallomics, offer opportunities to investigate metal(loid)–immune interactions at a systems level. However, their application in this field is still limited and requires integration into well-designed human studies.

### 9.4 Translational challenges from bench to bedside

Bridging the gap between mechanistic discoveries and clinical application remains a major challenge. Most preclinical studies rely on acute or high-dose exposures that do not reflect chronic, low-dose conditions typical of human environmental exposure, thereby limiting translational relevance. In addition, conventional experimental models often fail to capture interindividual variability in metabolism, genetic background, comorbidities, and concurrent treatments.

Clinical evidence linking metal(loid) exposure to immune checkpoint regulation and immunotherapy outcomes is still limited. While certain elements, such as zinc, have been proposed as potential biomarkers of ICI response, these findings are primarily based on observational studies and require validation in large, prospective cohorts.

Another important limitation is the lack of standardized biomarkers for assessing metal(loid)-induced modulation of immune checkpoint pathways in humans, which hinders clinical translation and trial design.

### 9.5 Clinical perspectives and future research priorities

From a clinical perspective, dysregulation of systemic metal(loid) ion homeostasis has emerged as a potentially important determinant of ICI outcomes, but the strength of evidence varies substantially across metal(loid)s and study types. At present, the most direct human evidence concerns trace-element biomarkers rather than interventions. Although mechanistic and preclinical evidence supports a plausible role for metal(loid)s in immune checkpoint regulation, direct human evidence remains limited, and most clinical data are observational and should currently be regarded as exploratory. Notably, human studies have primarily evaluated circulating trace-element levels or environmental metal exposure in relation to cancer outcomes, immune-related biomarkers, or ICI responses, rather than directly demonstrating causal modulation of PD-1/PD-L1 signaling by metal(loid)s in patients.

Among essential trace elements, zinc currently has the most direct human evidence in the context of ICI therapy. For example, higher serum zinc levels during ICI treatment have been associated with improved overall survival in observational studies, supporting its potential role as a predictive biomarker that requires further validation (55). However, this evidence remains exploratory because it is based on observational data, may be influenced by nutritional status, systemic inflammation, tumor burden, dietary supplementation, and treatment-related changes, and has not yet been validated in large independent prospective cohorts. Therefore, serum zinc should be considered a candidate biomarker rather than a clinically validated predictor of ICI response.

Human evidence for iron is also clinically suggestive but not definitive. Low serum iron has been associated with poorer outcomes in patients receiving PD-1 inhibitors for advanced gastric cancer. This finding is biologically plausible given the role of iron in T-cell metabolism and antitumor immune responses. Nevertheless, serum iron is strongly affected by inflammation, anemia, nutritional status, liver function, and tumor-associated cachexia. Thus, current iron-related findings should also be interpreted as exploratory and hypothesis-generating.

For copper, strong preclinical evidence indicates that intratumoral copper can modulate PD-L1 expression and tumor immune evasion. Copper-lowering approaches have shown promise in preclinical studies and early-phase clinical investigations, including combination strategies with immunotherapy, but their clinical efficacy and safety remain to be established (56). However, direct clinical evidence linking copper status to PD-1/PD-L1 expression or ICI response remains insufficient. Copper-related measures therefore represent promising but unvalidated translational candidates.

Beyond single-element supplementation or chelation strategies, traditional Chinese medicine (TCM) formulations, which may contain complex combinations of essential trace elements and bioactive compounds, represent a potentially underexplored therapeutic avenue. Sanshuibaihu decoction (SSBH), a traditional formula that has been clinically used for decades in the management of inflammatory disorders, has been reported to suppress NF-κB activation and p38 mitogen-activated protein kinase phosphorylation through its mineral- and animal-derived constituents, which may contain biologically active metal elements (57). These findings provide a preliminary rationale for further investigation of TCM-derived metal-modulating strategies as potential adjuvants to ICI-based therapies.

By contrast, for toxic metal(loid)s, the available evidence remains largely preclinical or indirect, and mechanistic findings should not be interpreted as sufficient support for clinical biomarker use or therapeutic intervention. Human studies mainly support broader associations between these elements (including arsenic, chromium, nickel, cadmium, lead, and mercury) and cancer risk, systemic inflammation, organ toxicity, or immune dysfunction, but direct clinical evidence connecting these exposures to immune checkpoint dysregulation or ICI outcomes is sparse. Their relevance to checkpoint-based immuno-oncology therefore remains largely indirect and requires prospective validation with standardized exposure assessment and immune profiling.

Intervention strategies aimed at modulating metal(loid) homeostasis should be approached cautiously. For zinc and selenium, a more appropriate near-term clinical strategy is correction of documented deficiency rather than empirical high-dose supplementation. Excessive zinc intake may cause adverse effects and disrupt copper homeostasis, with a tolerable upper intake level of 40 mg/day (58). Similarly, selenium supplementation remains investigational, with a tolerable upper intake level of 400 μg/day, and excessive intake may lead to toxicity (59, 60).

Looking forward, research priorities include standardized exposure assessment, development of longitudinal cohorts, integration of multi-omics approaches, and validation of metal(loid)-related biomarkers in immunotherapy settings. In the longer term, biomarker-guided intervention strategies may provide opportunities for precision immuno-oncology.

Taken together, no metal(loid)-related biomarker can currently be considered clinically validated for guiding ICIs. The strongest available human evidence supports exploratory associations for zinc and iron, whereas copper and toxic metal(loid)s remain largely supported by preclinical or indirect human evidence. Future studies should incorporate longitudinal exposure assessment, tumor and peripheral immune profiling, treatment-response endpoints, and adjustment for nutritional, inflammatory, environmental, and socioeconomic confounders.

Overall, coordinated multidisciplinary efforts are required to bridge environmental toxicology and cancer immunology and to translate mechanistic insights into clinically actionable strategies.

## 10 Conclusion

This review systematically examines the impact of metal(loid) exposure on immune checkpoint regulation and the underlying molecular mechanisms. Current evidence indicates that metal(loid) exposures can modulate immune checkpoint molecules through multiple pathways, including TIME remodeling, regulation of immune cell function, ROS-mediated immune checkpoint modulation, hypoxia signaling-mediated PD-L1 regulation, the JAK/STAT3 core immune signaling pathway, and tumor suppressor signaling and protein homeostasis regulation. Collectively, these findings suggest that metal(loid)-induced perturbation of immune checkpoint pathways may contribute to systemic adverse health consequences, including impaired antitumor immune responses and multi-organ effects.

Future studies should prioritize longitudinal cohort designs integrating multi-omics approaches—such as epigenomics, transcriptomics, proteomics, and metabolomics—to investigate interactions between metal(loid)s and immune checkpoints in susceptible populations, including occupationally exposed individuals and pediatric cohorts. Such investigations may help identify additional predictive biomarkers beyond the conventional PD-L1 tumor proportion score (TPS). Preclinical evaluation of combination strategies, such as metal(loid)-chelating agents in conjunction with ICIs, may inform approaches to overcoming ICI resistance, particularly as emerging metal(loid)-associated proteomic signatures may influence efficacy of ICIs.

Clinically, future prospective immunotherapy studies could incorporate standardized measures of internal metal(loid) burden as exploratory covariates, together with immune profiling and treatment-response endpoints. Such designs would help determine whether metal(loid)-related biomarkers provide independent predictive value beyond established factors such as tumor PD-L1 expression, tumor mutational burden, performance status, systemic inflammation, and nutritional status.

However, several limitations should be acknowledged. Most evidence is derived from preclinical models, limiting direct translation to humans. Accurate quantification of *in vivo* exposure is complicated by confounding factors, including co-exposure to multiple metal(loid)s, dietary influences, and genetic susceptibility, which complicate causal inference. Integration of immuno-oncology with nutritional and environmental toxicology may advance precision medicine approaches by supporting exposure-reduction strategies as a public health measure and by leveraging nutritional-toxicological evidence to guide therapeutic development. Future research should also prioritize tumor-specific and exposure-specific stratification to better define how different metal(loid)s interact with distinct TIME and influence immunotherapy outcomes.

## Glossary

ATO: arsenic trioxide
As₄S₄: tetraarsenic tetrasulfide
AT2: alveolar epithelial type 2
BTLA: B and T lymphocyte attenuator
CaCrO₄: calcium chromate
CDK4/6: cyclin-dependent kinase 4/6
CRC: colorectal cancer
CTLA-4: cytotoxic T-lymphocyte-associated protein 4
CuCl₂: copper(II) chloride
CUL3: Cullin 3
DC: dextran-catechin
EGFR: epidermal growth factor receptor
ESCC: esophageal squamous cell carcinoma
FOXP3: forkhead box P3
HIF-1α: hypoxia-inducible factor 1 alpha
HRE-4: hypoxia response element 4
ICB: immune checkpoint blockade
ICI: immune checkpoint inhibitor
IFN-γ: interferon gamma
IL: interleukin
ITIM: immunoreceptor tyrosine-based inhibitory motif
ITSM: immunoreceptor tyrosine-based switch motif
JAK: Janus kinase
LAG-3: lymphocyte activation gene 3
LC: Langerhans cell
lncRNA: long noncoding RNA
LUAD: lung adenocarcinoma
MDSC: myeloid-derived suppressor cell
miRNA: microRNA
MoLC: monocyte-derived Langerhans cell
NF-κB: nuclear factor kappa B
NFAT1: nuclear factor of activated T cells 1
NKG2D: natural killer group 2D
NSCLC: non-small cell lung cancer
OSCC: oral squamous cell carcinoma
PD-1: programmed cell death protein 1
PD-L1: programmed death-ligand 1
PD-L2: programmed death-ligand 2
ROS: reactive oxygen species
SeNPs: selenium nanoparticles
SHP-1: Src homology region 2 domain-containing phosphatase-1
SHP-2: Src homology region 2 domain-containing phosphatase-2
SIRT1: sirtuin 1
STAT3: signal transducer and activator of transcription 3
TAM: tumor-associated macrophage
TCR: T-cell receptor
TEPA: tetraethylenepentamine
TGF-β: transforming growth factor beta
Th: T helper
Th1: T helper 1
TIGIT: T-cell immunoreceptor with immunoglobulin and immunoreceptor tyrosine-based inhibitory motif domains
TIME: tumor immune microenvironment
TIM-3: T-cell immunoglobulin and mucin-domain containing 3
TNF: tumor necrosis factor
TNF-α: tumor necrosis factor alpha
TPS: tumor proportion score
TRAF3: tumor necrosis factor receptor-associated factor 3
Treg: regulatory T cell
Tyr705: tyrosine 705
USP15: ubiquitin-specific peptidase 15
ZnC: zinc L-carnosine
ZnSO₄: zinc sulfate.
